# Prevalence and Type Distribution of Human Papillomavirus Recovered from the Uterine Cervix of Nigerian Women: A Systematic Review and Meta-Analysis

**DOI:** 10.31557/APJCP.2020.21.10.2837

**Published:** 2020-10

**Authors:** Auwal Idris Kabuga, Ahmad Nejati, Amanuel Godana Arero, Somayeh Jalilvand, Talat Mokhtari-Azad, Shirin Shahbazi Sighaldeh, Umma Hassan Wali, Shohreh Shahmahmoodi

**Affiliations:** 1 *Department of Virology, School of Public Health, Tehran University of Medical Sciences, Tehran, Iran. *; 2 *Department of Medical Microbiology and Parasitology, Faculty of Clinical Sciences, College of Health Sciences, Bayero University, Kano, PMB 3011 Kano State, Nigeria. *; 3 *Students’ Scientific Research Center, Tehran University of Medical Sciences, Tehran, Iran. *; 4 *Department of Reproductive Health, School of Nursing and Midwifery, Tehran University of Medical Sciences, Tehran, Iran. *; 5 *Food Microbiology Research Center, Tehran University of Medical Sciences, Tehran, Iran. *

**Keywords:** Human papillomavirus, cervical cancer, risk factors, meta-analysis, Nigeria

## Abstract

**Background:**

Infection with an oncogenic type of human papillomavirus is a prerequisite for the development of precancerous cervical lesions and its subsequent progression to cervical cancer. With an alarming increase in the detection of other suspicious papillomavirus genotypes in both healthy and women with cervical lesions, there is a need for comprehensive data on cervical papillomavirus infection to address cervical cancer and other associated disease burden, especially in Sub-Sarahan Africa, where the bulk of the problem exists. The present study was conducted to develop comprehensive data on the prevalence and circulating genotypes of human papillomavirus in various risk categories in Nigeria.

**Methods:**

A systematic review and meta-analysis of peer-reviewed publications on cervical papillomavirus infection were performed. Relevant data were extracted from eligible studies published in PubMed, Web of Science, Embase, Scopus, and Google Scholar, from inception to July 31, 2019. The random-effect model was used to estimate the pooled prevalence. We identified 327 potential studies and pooled data from 18, involving 5697 women aged 15-86 years.

**Results:**

The overall pooled prevalence of cervical papillomavirus infection was 42% (95%CI: 30-54%) in the general population and 37% (95%CI: 25-50%) among women living with HIV/AIDs, with the predominance of genotypes 16, 18, 31, 35, 52, 58 and 45. The highest prevalence was observed in teenagers and young adults and the second peak in women 50 years and above.

**Conclusion:**

The prevalence of cervical human papillomavirus infection is cumulatively high in Nigeria and HIV is a strong co-factor. We, therefore, strongly recommend the co-screening of human papillomavirus and cervical cancer and integration of the intervention strategy into the existing HIV-care guideline in Nigeria.

## Introduction

The link between human papillomavirus (HPV) and cervical cancer is one of the highest identified in human cancerology, with almost all cases attributed to sexually acquired oncogenic genotypes, qualifying HPVs second after cigarette smoking as a risk factor for human cancers. Cervical cancer is considered as the fourth female cancer (Bray et al., 2018; Ferlay et al., 2019) and a common cause of cancer deaths in many parts of Sub-Saharan Africa. Cervical cancer is the second leading cause of cancer mortality in Nigeria (Ferlay et al., 2019). 

Following the establishment of the Expanded Program on Immunization (EPI) in 1974, the world has recorded a great success in controlling many infectious diseases; the eradication of smallpox and a significant reduction in the burden of many but especially viral diseases, like poliomyelitis, measles, and rabies (Levine et al., 2011). Similarly, HPV-related cervical cancers and hepatitis B virus-induced liver cancers are effectively prevented using appropriate reliable vaccines. With effective screening and vaccination, every woman can be protected against cervical cancer.

In Nigeria, there is poor awareness about HPV and its relationship with cervical cancer (Audu et al., 2014; Oluwasola et al., 2019) and no standardized data on HPV prevalence. This indicates that cervical cancer is neglected in the country, in terms of screening, prevention, and vaccination. Considering the population of women who are at risk of developing cervical cancer (over 50.3 million) in Nigeria, a country-specific HPV type distribution study is imperative. We conducted this systematic review and meta-analysis to provide comprehensive and representative data on prevalence, type distribution, and determinants of cervical HPV infection. 

## Materials and Methods

This study was conducted in compliance with the PRISMA guidelines and reports relevant to the outcome of interest included in the quantitative synthesis (Moher et al., 2009). The outcome of interest includes the prevalence of cervical HPV infection, risk factors, and circulating genotypes in Nigeria. 

*Search strategy and data sources*

We performed a comprehensive search of databases to identify all relevant articles published on cervical HPV infection, including prevalence, risk factors, and distribution of the circulating genotypes in Nigeria. Databases explored were PubMed, Web of Science, Embase, Scopus, and Google Scholar, from inception to July 31, 2019, when the search was last updated. References of all selected studies were further screened for studies connected to HPV infection in the country.

*Inclusion and exclusion criteria*

Studies of any design published in English Language and peer-reviewed journals were included, with emphasis on adequate samples and enough data to determine the prevalence, distribution of HPV types, and determinants of cervical HPV infection. Only studies that checked for at least two clinically relevant HPV genotypes (e.g. 16 and 18) from the cervical specimen, using a molecular-based approach (e.g. PCR, HC, LiPA25) with a sample size of at least 30 were included in the final data synthesis. Thirteen studies were excluded based on duplication of data or study population (Schnatz et al., 2008; Clarke et al., 2011; Gage et al., 2012a; Akarolo-Anthony et al., 2013; Famooto et al., 2013; Musa et al., 2013; AdegbesanOmilabu et al., 2014; Akarolo-Anthony et al., 2014; Dareng et al., 2016; Manga et al., 2016; Nyengidiki and Athanasius, 2016; Adebamowo et al., 2018; Nyengidiki and Oranu, 2018), and two due to inadequate sample size or unclear results (Kolawole et al., 2015; Kolawole et al., 2016). Two studies comparing cervical sampling methods (Modibbo et al., 2017; Ajenifuja et al., 2018), and a study with no information about HPV types checked (Nejo et al., 2018) were further excluded. Finally, 18 studies were included in the data synthesis. 

*Data extraction and quality assessment*

Pre-designed excel data extraction was performed and the following data extracted; the name of the first author, publication year, the period of study, the geopolitical zone where the study was conducted, study site, age range, mean/or median age of the participants, the total number included, number positive, classification of cytological findings, method of detection, genotypes targeted for and predominant types identified as well as risk factors. Newcastle Ottawa scale for cross-sectional studies was used to assess the quality of the included studies.

*Statistical analysis*

A meta-analysis using the DerSimonian and Laird random effect model was performed to calculate the pooled prevalence estimates. The heterogeneity of the studies was assessed using Cochran’s Q *χ2* statistic and Higgins’s method I^2^-statistic. Cochran’s Q statistics (P<0.1) indicates a statistically significant variation among the studies, while I^2^-statistics quantifies the proportion of variance explained between studies heterogeneity. Meta-regression analysis was performed to check for a potential source of heterogeneity by considering the year of publication. Both funnel plot symmetry and Egger’s regression asymmetry test and Begg rank correlation methods were used to evaluating the presence of publication bias. The outcome of sensitivity evaluation confirmed that our findings were robust and not one-study dependent. The pooled prevalence estimates of the infection range from 39% (95%CI: 29-49%) to 44% (95%CI: 32-56%) after single-study removal. We conducted subgroup analyses by administration divisions to provide summary estimates for various risk categories and methods of detection. For all analyses, Stata software (StataCorp, TX USA, version 14.0) was used and a two-tailed P<0.05 considered statistically significant.

## Results

**Figure 1:**
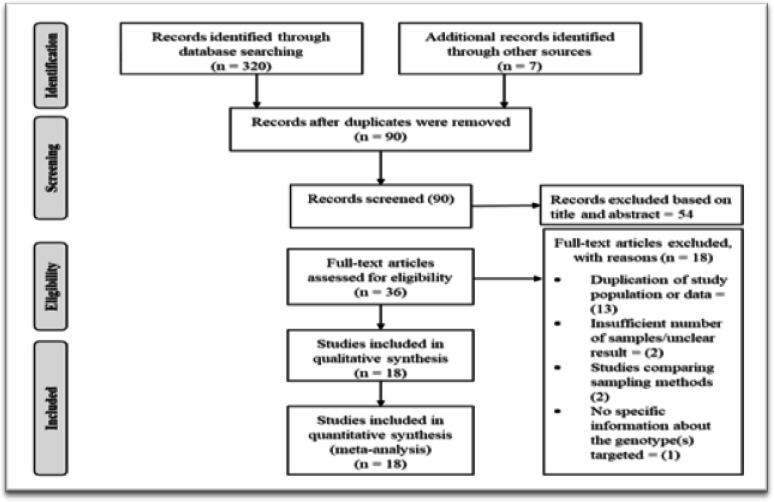
PRISMA Flowchart Showing Selection of the Eligible Studies on Cervical Papillomavirus Infection in Nigeria, 2019

The process of article selection is shown in Figure 1. Most of the studies were accomplished using PCR of DNA extracted from cervical samples, collected from 5,697 women aged 15-86 years from the six geo-political zones. 

*Characteristics of the included studies*

All included studies were cross-sectional in design and majority hospital-based and employed PCR techniques. Looking at the oncogenic and low-risk HPV types from the cervices of women aged 15 to 86 years, a wide range of prevalence (4.9%-85.6%) was observed. The included studies were carried out from June 1999 to May 2017 and published between 2004 to 2019 as shown in Table 1. 

*Heterogeneity and publication bias*

**Figure 2:**
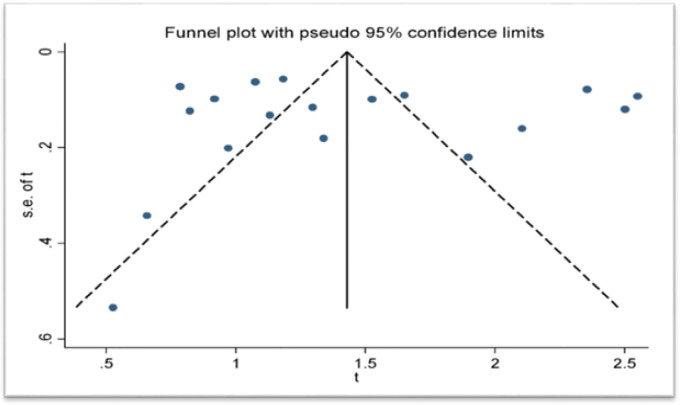
Funnel Plot of Arcsine Transformed Prevalence Estimates (t) of HPV Recovered from the Uterine Cervix of Nigerian Women, 2019

Studies were evaluated for potential publication bias by qualitative and quantitative approaches and the funnel plot showed the absence of publication bias (Figure 2). Absence of publication bias was also confirmed by Begg’s rank correlation test (p = 0.705) and Egger’s weighted regression test (bias coefficient = 1.1595 (95%CI: -5.970982 to 8.289982; p = 0.735). Given a significant variation among the studies (I^2^ = 99.2, p <0.01), random effects model was applied to estimate the DerSimonian and Laird’s pooled effect. 

*Overall pooled prevalence of cervical papillomavirus infection in Nigeria*

**Figure 3:**
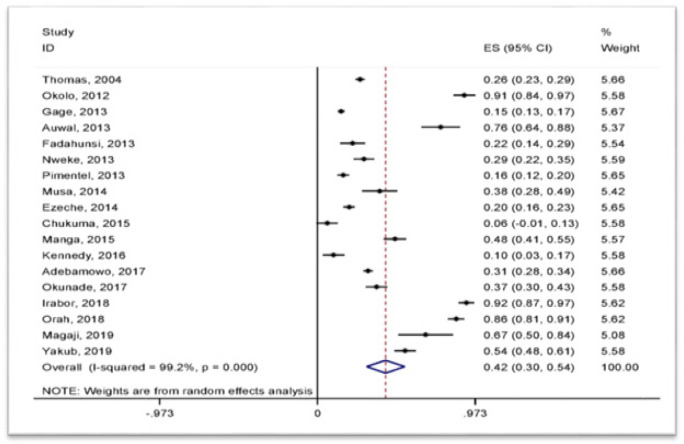
Forest Plots Indicating a Pooled Prevalence of Cervical HPV Infection in Nigeria, 2019. The overall pooled prevalence was 42% (95%CI: 30-54%).

The prevalence of cervical HPV infection was determined as the proportion of HPV positive cases for any type expressed in percentage, giving an estimated pooled prevalence of 42% (95%CI: 30-54%) (Figure 3). As indicated in Table 2, the prevalence varies with geopolitical zone, population sub-unit, detection method, and histologic classification of cervical findings. 

*Estimated pooled prevalence in HIV positive women (HIV-HPV coinfection)*

A cohort of studies conducted among HIV positive women was included and we performed subgroup analysis by HIV positivity, giving a pooled prevalence of 37% (95%CI: 25-50%). HIV was found to exert a strong effect on the acquisition of cervical HPV infection in Nigeria [OR = 2.34 (95%CI = 1.36-4.02%): p<0.01], as shown in Table 4. 

*Age-specific prevalence of cervical HPV infection in Nigeria*

The prevalence of cervical HPV varies significantly among different age groups in Nigeria and shows bi-modal age peaks. The highest prevalence was estimated among teenagers 45% (95%CI: 27-63%) and young adults 44% (95%CI: 13-75%) who were at the climax of their sexual activity, then a second peak 43% (95%CI: 5-81%) in women 50 years and above. 

*HPV type-specific prevalence*

Majority of the included studies paid emphasis on cancer-causing genotypes (Table 1). Although commonly reported types varied slightly by the study, HPV type 16 predominates with an overall pooled prevalence of 27% (95%CI: 18-35%), followed by type 18, 31, 35, 52, 58, and 45. HPV-16 was predominantly recovered in HGSILs and cervical cancer with a prevalence of 51% (95%CI: 33-69%), followed by genotype 18, 45, and 35. Predominant genotypes in HIV positive were 35, 16, 58, 52, 45, 31, 33 and 18 (Table 3). 

*Association between risk factors and cervical HPV infection in Nigeria*

Some included studies evaluated the potential factors thought to be associated with the acquisition of HPV infection. Our analyses indicated a statistically significant association with multiple sexual partners [OR = 1.46 (95%CI: 1.19-1.8); p<0.01] and HIV infection [OR = 2.34 (95%CI: 1.36-4.02); p<0.01]. As indicated in Table 4, other indicators of sexual/reproductive behavior, literacy, place of residence, and CD4+ T-cell count showed various forms of association.

*Meta-regression analysis*

**Figure 4:**
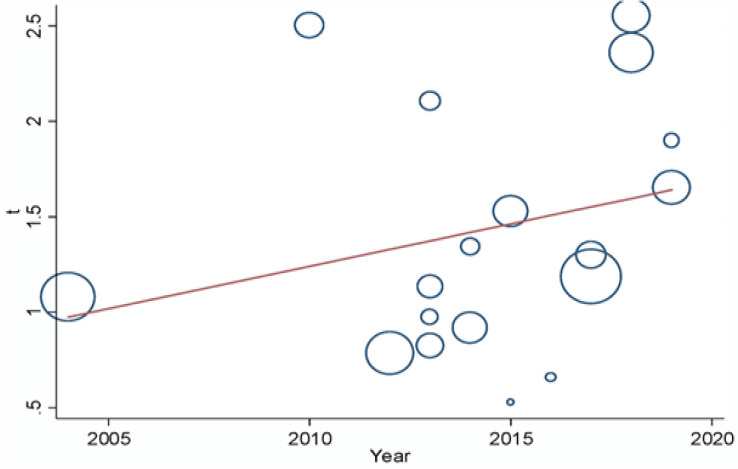
Meta-Regression of Cervical HPV Infection by Year, Nigeria, 2019

Univariate Meta-regression analysis was performed to assess the pattern of cervical HPV infection among the included population by year of publication. Although graphically, there is evidence of an increase in the trend of the infection from 2004 to 2019, the increment has no statistical effect (coefficient = 0.0443924: p = 0.292) (Figure 4).

**Table 1:** Summary of Studies Included in Final Data Synthesis

Study (year)	R	S	Study period	Age range	µ or med age	No tested	No pos.	Assay	HPV types checked	Ref.
Thomas, 2004	SW	C	Jun 1999-	≥15		932	245	PCR	6,11,16,18,26,31,33,34,35,39,40,42,43,44,45,51,52,53,54,	(Thomas et al., 2004)
			Apr-00						55,56,57,58,59,61,66,68,70,71,72,73,81,82,83,84,CP6108	
Okolo, 2010	SW	H	2004-2006	31-80	55	75	68	PCR	16,18,35,45,51,56	(Okolo et al., 2010)
Gage, 2012	SW	C			45	1282	188	PCR	16,18,31,33,35,39,45,51,52,56,58,59,68	(Gage et al., 2012b)
Auwal, 2013	NW	H	6 months		28	50	38	PCR	16,18	(Auwal et al., 2013)
Fadahunsi, 2013	SW	H	Mar-Feb 2011	18-68	42.9	111	24	HC	16, 18, 33, 35, 45,52,53,68	(Fadahunsi et al., 2013)
Nweke, 2013	SW	H	Aug 2011-Aug 2012		40.3	195	56	HC/	6,11,16,18,31,33,35,39,44,45,51,52,53,56,58,	(Nweke et al., 2013)
								PCR	59,66,68,CP8304	
Pimentel, 2013	NC	H	2004-2008	19-85	36.2	401	64	HC	16,18,31,33,35,39,45,51,52,56,58,59,68	(Pimentel et al., 2013)
Ezeche, 2014	SW	RC	1 year	18-81	37	515	101	PCR	16,18,31,33,35,39,45,51,52,56,58,59,68	(Ezechi et al., 2014)
Musa, 2014	NC	H	May 2012-Jun 2013		32.4/32	78	30	HC	16,18,31,33,35,39,45,51,52,56,58,59,68	(Musa et al., 2014)
Chukuma, 2015	SE	H		18-64		50	3	PCR	6,11,16,18	(Chukwuma et al., 2015)
Manga, 2015	NE	H	Aug 2013-May 2014		39.6	209	100	PCR	16,18,31,33,35,38,45,56,58,82,KC5	(Manga et al., 2015)
Kennedy, 2016	SS	H	Aug-Dec 2014	16-62	39	80	8	PCR	16,18,31,35	(Kennedy et al., 2016)
Adebamowo, 2017	NC	H	2012-2014			962	300	SPF10/ LIPA25	6,11,16,18,31,33,34,35,39,40,42,43,45,51,52, 53,54,56,58,59,66,68/73,70,74,HPV-U	(Adebamowo et al., 2017)
Okunade, 2017	SW	H	6 months	20-63	36.1	200	73	PCR	16,18,31,33,35	(Okunade et al., 2017)
Irabor, 2018	SS	H	Jan 2009-Dec 2014	32-75	48.6	123	113	PCR	At least 54 genotypes were targeted	(Irabor et al., 2018)
Orah, 2018	SW	H	2000-2004	22-86		187	160	SPF10/	6,11,16,18,31,33,35,39,40,45,51,52,56,58,	(Orah and Banjo, 2018)
								LIPA25	59,66,68	
Magaji, 2019	NW	H	2015			30	20	PCR	16,18,31,45	(Magaji et al., 2019)
Yakubu, 2019	NC	H	Aug 2016-May 2017	20-50		220	119	PCR	6,11,16,18,31,33,35,39,40,41,42,43,44,45,51,52,56,66	(Yakub et al., 2019)

R, Geographical region; SW, South West; NC, North Central; SS, South-South; NW, North West; SE, Southeast; NE, North East; S, Study site; H, Hospital; C, Community, RC, Research center; Mean; PCR, Polymerase chain reaction; HC, Hybrid capture, µ= mean.

**Table 2:** Subgroup Analysis of cervical HPV Infection in Nigeria, 2019

Characteristic	Category	No of studies	Pooled prevalence (95%CI)	I^2^(P-value)	Difference b/w subgroup χ^2^ test p-value
Overall	-	18	42 (30-54)	99.2 (<0.01)	-
	SW	8	40 (23-58)	99.3 (<0.01)	
	NC	4	35 (20-49)	97.3 (<0.01)	
Zone	SS	2	51 (-29-131)	99.7 (<0.01)	
	NW	2	73 (63-83)	0 (0.38)	99.2 (<0.01)
	SE	1	6 (1-13)	-	
	NE	1	48 (41-550	-	
Excluding WLWHA	-	15	42 (28-56)	99.3 (<0.01)	-
	SW	8	38 (20-56)	99.4 (<0.01)	
	NC	2	19 (13-24)	77.3 (0.04)	
Zone	SS	2	51 (-29-131)	99.7 (<0.01)	99 (<0.01)
	NW	2	73 (63-83)	0 (0.38)	
	NE	1	47 (40-54)	-	
WLWHA	-	8	37 (25-50)	95.1 (<0.01)	
	Normal	7	27 (19-36)	95.6 (<0.01)	
Histology	Abnormal	9	66 (52-81)	94.1 (<0.01)	
	Ca cervix	3	87 (83-91)	0 (0.53)	
	CIN-3	2	57 (21-94)	0 (0.81)	
	<30	6	45 (27-63)	93 (<0.01)	
Age (years)	30-39	5	44 (13-75)	97.3 (<0.01)	
	40-49	5	38 (8-67)	78.6 (<0.01)	
	≥50	5	43 (5-81)	99.1 (<0.01)	
Method of Detection	PCR	13	43 (29-58)	99.2 (<0.01)	99.2 (<0.01)
	HC	3	24 (13-36)	87.2 (<0.01)	
	SPF10/LIPA25	2	58 (5-112)	99.7 (<0.01)	

WLWHA, Women living with HIV/AIDs; SW, South West; NC, North Central, SS, South-South; NW, North West; SE, southeast; NE, North East; Ca cervix, cervical cancer; CIN, cervical intraepithelial neoplasia; PCR, polymerase chain reaction; HC, hybrid capture assay; I^2^, heterogeneity; CI, confidence interval.

**Table 3:** Type specific Prevalence of Cervical HPV Infection in Nigeria, 2019

Genotype	Overall			WLWHA			Cervical cancer & HGSILS		
	No of studies	Pooled prevalence (95%CI)	I^2^%(P)	No of studies	Pooled prevalence (95%CI)	I^2^%(P)	No of studies	Pooled prevalence (95%CI)	I^2^%(P)
16	14	27 (18-35)	95.9 (<0.01)	3	15 (5-24)	83.5 (<0.01)	3	51 (33-69)	85.3 (<0.01)
18	14	23 (15-31)	95.2 (<0.01)	3	8 (3-12)	61.7 (0.07)	3	14 (7-21)	52.1 (0.12)
31	11	15 (9-21)	94.6 (<0.01)	3	10 (1-19)	90.9 (<0.01)	3	3 (0-5)	34.3 (0.22)
33	9	4 (2-6)	70.8 (<0.01)	3	7 (1-12)	79. (0.01)	3	1 (0-3)	0 (0.76)
35	12	11 (7-15)	81.7 (<0.01)	3	19 (14-24)	18.4 (0.29)	3	8 (1-14)	64 (0.06)
39	6	2 (1-3)	12.3 (0.34)	3	2 (0-3)	0 (0.44)	-	-	-
45	10	6 (4-8)	55.3 (0.02)	3	8 (2-13)	71.8 (0.03)	3	9 (5-13)	14.5 (0.31)
51	7	5 (2-7)	86 (<0.01)	3	5 (-1-12)	86.2 (<0.01)	3	2 (1-5)	30.8 (0.24)
52	7	9 (5-14)	94.5 (<0.01)	3	11 (-2-24)	94.6 (<0.01)	-	-	-
56	9	4 (3-6)	60.9 (0.01)	3	4 (2-6)	0 (0.67)	3	3 (2-7)	58.9 (0.12)
58	6	8 (3-13)	92.7 (<0.01)	3	10 (0-21)	75.2 (0.045)	-	-	-
59	5	2 (1-3)	46.5 (0.11)	3	4 (0-7)	50.9 (0.15)	-	-	-
68	5	4 (1-6)	76.3 (<0.01)	2	3 (1-6)	0 (0.54)	-	-	-
Other types	5	8 (3-13)	80.5 (<0.01)	-	-	-	-	-	-

WLWHA, women living with HIV/AIDs; Other types include HPV-53, 70 & 82; HGSILs, High grade squamous intraepithelial lesions; I^2^, Heterogeneity; P, p-value; CI, Confidence interval.

**Table 4:** Subgroup Analysis by the Risk Factors

Risk factor	Category	No of studies	Pooled OR (95%CI)	I^2 ^(p-value)	Z-test p-value
Age (years)	<30	5	1.44 (0.88-2.35)	47.9 (0.10)	0.14
	≥30		0.69 (0.43-1.13)		
Age at sexual debut	<18	2	1.47 (0.72-2.99)	77 (0.04)	0.29
	≥18		0.68 (0.33-1.38)		
No of sexual partners	>1	7	1.46 (1.19-1.8)	11.5 (0.34)	<0.01
	1		0.68 (0.56-0.84)		
Parity	>4	5	1.43 (0.75-2.72)	81.9 (<0.01)	0.28
	≤4		0.70 (0.37-1.33)		
Husband having more than one sex partner	Yes	3	1.38 (0.98-1.94)	0 (0.54)	0.07
	No		0.73 (0.51-1.02)		
History or evidence of STIs other than HIV	Yes	3	3.05 (0.95-9.73)	87 (<0.01)	0.02
	No		0.33 (0.1-1.06)		
Use of barrier contraceptive	Never use	3	1.19 (0.81-1.75)	0 (0.64)	0.37
	Ever use		0.84 (0.57-1.23)		
Use of hormonal/oral contraceptive	Ever use	4	0.98 (0.7-1.37)	0 (0.55)	0.9
	Never use		1.02 (0.73-1.43)		
Place of residence	Urban	3	1.02 (0.46-2.26)	61.2 (0.08)	0.96
	Rural		0.98 (0.44-2.18)		
Literacy level	Low	4	1.34 (0.63-2.86)	69.6 (0.02)	0.45
	High		0.75 (0.35-1.6)		
HIV status	Positive	5	2.34 (1.36-4.02)	75.6 (<0.01)	<0.01
	Negative		0.43 (0.25-0.73)		
CD4 cell count	<500	2	16.46 (0.2-1378.6)	79.1 (0.03)	0.22
	≥500		0.06 (0-5.09)		

OR, odds ratio; CI, confidence interval; I^2^, heterogeneity; STI, sexually transmitted infection; IUCD, intrauterine contraceptive device.

## Discussion

Epidemiological studies on prevalence and distribution of HPV infection are not just important in evaluating the impact of the existing cervical cancer screening and HPV vaccination programs but also provides the opportunity to explore other relevant tools to combat cervical cancer and other HPV related diseases. In Sub-Saharan Africa, there is significant regional variation in the incidence of HPV infection and cervical cancer, and the highest burden observed in the East and Southern parts (De Vuyst et al., 2013). In Nigeria, data on cervical HPV infection is highly variable and incomplete. While studies conducted in hospitals remained the major source of data, in most cases the studies are designed by convenience of the researchers. Our study revealed a high prevalence of 42% (95%CI: 30-54%). However, the prevalence is lower than that of Rwanda (Ngabo et al., 2016), Zimbabwe (Dube Mandishora et al., 2017), Angola (de Almeida Damião et al., 2016), Gabon (Assoumou et al., 2016), South Africa (Ebrahim et al., 2016; Mbulawa et al., 2018), Swaziland (Ginindza et al., 2017) and Pakistan (Siddiqa et al., 2014)¬, but higher than that of Cameroon (Doh et al., 2017), Ethiopia (Leyh-Bannurah et al., 2014), Gambia (Camara et al., 2018), The Demographic Republic of Congo (Mutombo et al., 2019), Chad (Bouassa et al., 2019), Bangladesh (Nahar et al., 2014), Tunisia (Guettiti et al., 2014), Islamic Republic of Iran (Kesheh and Keyvani, 2019), India (Sabeena et al., 2017), Nepal (Shakya et al., 2017) and China (Su et al., 2019). The fact that Nigeria is cosmopolitan with diverse ethnic groups and cultural practices, which in practice affect disease transmission and spread, may account for the high prevalence observed. Using different age categories, subgroup analysis demonstrated a bi-modal age-specific peak as observed in previous studies (Castle et al., 2005; De Sanjosé et al., 2007; Mutombo et al., 2019; Su et al., 2019). This finding is not surprising considering the role of intergenerational sexual relationships in fueling the spread of sexually transmissible infections (Oyediran et al., 2011; Baussano et al., 2013; Mavhandu-Mudzusi et al., 2014; National Bureau of Statistics and Fund, 2017). In intergeneration marriage (a common practice in many parts of Nigeria), an infected older man is likely to infect his young-lady-partner or acquire the infection and reintroduce to a woman who probably had and cleared the virus during her active sexual life. Perhaps, a young man having a sexual relationship with an infected older woman can acquire and pass the virus to his younger partner, and vice-versa. An HPV naïve adolescent woman, due to fragile nature of her system, can acquire the infection from infected co-wife(ves), since she is at crucial period of contacting many sexually transmitted infections. The highest prevalence of cervical HPV infection observed among adolescent and grown-up females matched with reports from other countries (Tricco et al., 2011; Bahmanyar et al., 2012; Ebrahim et al., 2016; Ginindza et al., 2017; Bouassa et al., 2019; Mutombo et al., 2019; Su et al., 2019). The results of a randomized control trial (Sankaranarayanan et al., 2009) suggests the reliability of PCR in detecting HPV DNA as used by majority of the included studies. Nevertheless, studies employing serology, hybrid capture, cytology and SPF10/LIPA25 should not be discourage as they provide clues and guide clinical decision-making. Reflecting on the genotypes diversity, the high prevalence of oncogenic types might suggest increasing tendency for developing cervical cancer and high burden in Nigeria. 

The fact that HIV positive women are more likely to acquire HPVs has been demonstrated (Wang et al., 2015; Menon et al., 2016; Obiri-Yeboah et al., 2017). HIV is thought to favor the persistence of cervical HPV infection, which in turn results in precancerous lesions, and potentiate progression of the lesions to cancer (Ahdieh et al., 2001; Palefsky, 2003; Strickler et al., 2005). Furthermore, the lack of treatment for HPV infection and increase life expectancy in HIV infected individuals (due to availability of antiretroviral drugs) intensify the risk of developing high-grade cervical lesions and carcinomas (Cañadas et al., 2010; Cohen et al., 2019). Although several researchers reported a higher prevalence of HPV in HIV positive women, the lower prevalence (37%) observed in this study might be due to a small number of HIV positive women included as evidenced by the 95%CI: 25-50%. However, the variation observed in HPV genotypes distribution coincides with findings of previous reports (Menon et al., 2016; Obiri-Yeboah et al., 2017), highlighting the necessity of strict monitoring of HPV infection in women living with HIV/AIDs in Nigeria. Also, the association of HPV infection with multiple sexual partners and other sexually transmitted infections, long periods of and recurrent exposure to HPV oncogens increase the likelihhod of developing premalignant lesions and cervical cancer (Doh et al., 2017). 

Moreover, immense disease burden and fragile health care system embedded in the broader context of poverty, underdevelopment, poor governance, political instability, internal conflicts, and insecurity of lives and properties might explain the increasing incidence of HPV related diseases in Sub-Saharan Africa and some Asian countries. With these constraints, it is nearly impossible to come up with a sustainable cytology-based cervical cancer prevention program, talkless of making the HPV vaccines available and affordable.

In conclusion, this systematic review and meta-analysis illustrated a high prevalence and variation in the distribution of cervical HPV infection in Nigeria and identified HIV as a significant cofactor. In addition to HPV genotypes 16 and 18, the study also identified a high burden of other oncogenic HPV variants that are not included in the first generation of HPV vaccines. The infections are mostly in teenagers and young adults and there is a second peak at the age of 50 years and above. Our findings suggested a comprehensive cervical cancer screening program, incorporating HPV testing as an additional triaging modality. To improve knowledge about HPV infection, prevention, and vaccine uptake, we recommend school-based campaigns and vaccination programs. There is also a need for incorporating HPV testing as part of the existing HIV prevention and treatment. 
